# Anthropometric Indicators and Immune Fitness: An Exploratory Online Survey Among Adults from Saudi Arabia

**DOI:** 10.3390/healthcare14081046

**Published:** 2026-04-15

**Authors:** Azzah S. Alharbi

**Affiliations:** 1 Clinical Microbiology and Immunology Department, Faculty of Medicine, King Abdulaziz University, Jeddah 21589, Saudi Arabia; asalharbi3@kau.edu.sa; 2 Special Infectious Agents Unit, King Fahd Medical Research Center, King Abdulaziz University, Jeddah 21589, Saudi Arabia

**Keywords:** obesity, immune fitness, anthropometric, BMI, underweight, obese

## Abstract

**Objectives**: Given the limitations of body mass index (BMI) as a metric and the lack of data on the relationship between various anthropometric indices of obesity and immune fitness (IF), this study aimed at exploring the possible association between various anthropometric indicators and the immune fitness among an adult sample of the Saudi population residing in Makkah. **Methods**: A structured self-reported questionnaire, with questions covering age, sex, anthropometric and immune fitness assessment data, was distributed online to a convenience sample of target population. The Immune Status Questionnaire (ISQ) was utilized to assess respondents’ IF over the past 12 months, while perceived momentary immune fitness (PMIF) was measured using a single-item scale. A total of 1135 responses were included in the study. **Results:** Overall, 530 male (46.7%) and 605 female (53.3%) respondents were included in the analysis. Of these, 478 (42.1%) had a normal BMI, and 343 (30.2%) were classified as overweight, 184 (16.2%) as obese, and 130 (11.5%) as underweight. Participants with reduced ISQ score (<6) were more likely to be underweight (*p* < 0.001), have a high weight-adjusted waist index (WWI) (*p* = 0.035), and exhibit an increased conicity index (C index) (*p* = 0.037) compared to those with an ISQ score ≥ 6. After controlling for age and sex, weight (*p* = 0.003), height (*p* < 0.001), and WWI (*p* = 0.01) were found to have significant correlations with past-year IF, while only height (*p* = 0.004) showed a significant positive correlation with PMIF. A multiple linear regression analysis revealed that sex and height and waist circumference (WC) were significant predictors of IF. Specifically, males and those who were taller had higher IF scores. Whereas individuals with high-risk WC values reported lower IF scores than those with low-risk WC. **Conclusions:** Sex (male) and anthropometric measures (lower WC, and taller height) were the most informative predictors of higher IF scores. The findings highlight the association between anthropometric measures and IF. A deeper understanding of these associations can inform the development of targeted interventions aimed at improving IF and overall health outcomes.

## 1. Introduction

Immune fitness (IF) describes the adequate functioning of the immune system and, more specifically, describes an individual’s capability to effectively trigger appropriate immunological responses against health threats [1]. Reduced IF plays a critical role in the emergence of both communicable and non-communicable diseases (NCDs), such as neoplasms, depression, neurodegenerative diseases, asthma, and cardiometabolic disorders. It can be a contributing factor or an outcome of these conditions [2,3,4]. Additionally, reduced IF significantly impairs mood, health perception, day-to-day activities, social interactions, and the quality of life (QoL) [5,6,7]. The economic burden of reduced IF related to chronic illness is expected to be substantial, considering both direct healthcare costs and lost productivity [3].

The reduced IF can result from genetic predisposition and individual attributes like age, gender, and existing health conditions. Still, it is also heavily influenced by lifestyle choices such as dietary habits, levels of physical activity, sleep quality, and chronic stress [8]. Obesity is a significant determinant of reduced IF among modifiable factors. It leads to chronic immune dysfunction, caused by impaired adipose-derived signaling, disturbed metabolism of lipids, metabolic stress and imbalances in the regulatory hormones of the immune cells, that eventually leads to an inflammation and compromises the immune surveillance, infection defense, and vaccine response [9]. Obesity was recognized as an independent risk factor for hospitalization and mortality associated with the 2009 H1N1 pandemic influenza [10]. Moreover, obese individuals experienced higher SARS-CoV-2 infection rates and were more likely to develop severe COVID-19 symptoms, elevated hospitalization rates, and notably higher mortality rates [11,12,13,14]. During the initial months of the COVID-19 pandemic, while many contracted SARS-CoV-2, the risk of severe illness and death varied among populations. Vulnerable subpopulations, such as the elderly and those with underlying chronic illnesses (particularly NCDs), experienced more severe symptoms and higher risks of hospitalization and death. It is due to impaired immune function characterized by obesity-induced chronic systemic low-grade inflammation [15]. Together, these findings underscore its significant role in shaping the progression of infections, prompting a need for further exploration of its connection with immune fitness.

Obesity is a rapidly expanding global health concern, and its prevalence has almost tripled worldwide since 1975. In 2022, it is estimated that approximately 16% of adults (890 million) and 160 million children and adolescents globally are impacted by obesity [16]. A recent study conducted in the Kingdom of Saudi Arabia (KSA), specifically in the Makkah region, reported the incidence of overweight and obesity among the population as 32.8% and 23%, respectively [17]. NCDs are extensively reported in KSA and account for 73% of all fatalities in the country [18]. Thus, poor IF could be a prevalent issue affecting the entire community.

Prior research has demonstrated that biomarkers can evaluate clinically relevant aspects of the immune system, such as cytokines [19]. Obesity-related measures were inversely linked to changes in cytokines; physical activity lowered inflammatory cytokines [20], and in obese children, physical exercise reduced pro-inflammatory cytokines and increased antimicrobial peptides [21]. Nevertheless, no biomarker fully captures the overall concept of IF. Thus, specific scales have been designed to subjectively evaluate IF, including the Immune Status Questionnaire (ISQ), which is accompanied by a 1-item rating of perceived momentary immune fitness (PMIF) [15,22,23]. PMIF reflects the subjective perception of individuals’ overall immune fitness at the time of assessment measured by a single-item rating scale (0 = very poor, 10 = excellent). While the ISQ is a multi-item, retrospective test that evaluates the overall immune fitness objectively over the last year [22], the IF scale offers a widely applicable, affordable, and non-invasive method for evaluating immune fitness, aiding in the identification of individuals at risk and facilitating early intervention. Obesity is commonly assessed using body mass index (BMI); however, BMI has its limitations [24,25,26].

There is a lack of knowledge about the association between IF and various adiposity-related anthropometric measures in Saudi population-based studies. Due to the population-specific demographic, nutritional and lifestyle factors, findings from other regions might not be directly applicable to the Saudi population. Given the importance of this understanding for public health and clinical practice, it is necessary to facilitate the development of more effective interventions in KSA. The present study aimed to (1) examine the associations between immune fitness and deviations from normal BMI categories, and (2) identify adiposity-related anthropometric indices beyond BMI that are most strongly associated with poor immune health in the Saudi population.

## 2. Methods

### 2.1. Design and Sample

The current cross-sectional investigation was conducted from December 2023 to February 2025. Ethical approval was obtained from the Research Ethics Committee at King Abdulaziz University (KAU) (reference number 495-22/1), and the study was conducted according to the principles outlined in the Declaration of Helsinki. Informed consent was obtained from all subjects and/or their legal guardian(s). A structured questionnaire, created using Google Forms, was distributed via various social media platforms, including Facebook, WhatsApp, and Instagram. The sampling technique was convenience-based. Those who gave their consent were included in the study. The survey targeted Saudi citizens and residents of the Makkah region who were 18 years old or older. This region was selected due to its high incidence of obesity among the local population [27]. It is also recognized as one of the top five areas in the KSA with the highest rate of obesity [28]. Participation in the survey was voluntary, and the confidentiality of all responses was maintained. The study’s objectives and rationale were clearly stated, and online consent was recorded at the beginning of the form. Access to the questionnaire was restricted to those who consented to participate, and the submission of multiple responses per participant was blocked. Participants with a history of medical issues compromising immunity or those on immunosuppressive medication were not included.

### 2.2. Questionnaire Content

Data were collected on age, gender, and self-reported anthropometric parameters (height, weight, and waist circumference). Self-reported anthropometric parameters have been shown to correlate significantly with measured values, making them useful in large-scale population surveys [29,30,31]. To improve accuracy, participants were given clear written instructions with illustrations and were encouraged to refer to recent medical records or take fresh measurements before completing the survey. This approach aligns with recent trends in large-scale epidemiological research, which increasingly relies on digital platforms to facilitate real-time, low-cost, and decentralized health surveillance as well as efficient monitoring of health indicators [32]. BMI, waist-to-height ratio (WC:Ht), conicity index (C index), and weight-adjusted waist index (WWI) measures of obesity were calculated. An individual’s body mass index (BMI) is calculated by dividing their weight by their height in meters squared. The resulting BMI values were divided into “four categories: underweight (BMI less than 18.5 kg/m^2^), normal weight (BMI 18.5 to 24.9), overweight (BMI 25 to 29.9), and obese (BMI 30 kg/m^2^)” [29]. Values of WC were divided into “three categories: low risk (WC < 80 cm for women and <94 cm for men), moderate risk (WC 80–87.9 cm for women and 94–101.9 cm for men), high risk (WC >88 cm for women and >102 cm for men)” [33]. Conicity index (C index) [34], and weight-adjusted waist index (WWI) [35] were measured as follows:

$$CI=\frac{waist\;circumference(m)\;}{0.109\times \sqrt{\frac{weight(kg)}{height(m)}}}$$

$$WWI=\frac{waist\;circumference(cm)}{\sqrt{weight(kg)}}$$

WWI, a new anthropometric index, was shown recently to provide a better indication of body composition and health risk independent of body size than conventional measures [36,37,38,39,40]. WWI values were divided also into “three categories: low risk < 10, moderate risk 10 to 11, high risk > 11”. Since there are no universal international guidelines for WWI, these thresholds reflect meaningful risk stratification observed in multiple cohorts [41,42].

The ISQ was used to evaluate respondents’ IF over the past 12 months. The ISQ consists of seven items that assess the frequency of sudden high fever, diarrhea, headaches, skin issues, aching muscles and joints, common colds, and coughs using a five-point Likert scale ranging from never to occasionally, regularly, often, and always. A total ISQ score of 0 to 10 was assigned, with a threshold of 6 indicating a decline in immune fitness during the previous year. This cut-off has been applied in earlier studies that have confirmed the ISQ’s reliability and validity [1,7,8,23,43,44]. Additionally, single-item questions were used to rate the PMIF and general health on a scale from 0 (worst) to 10 (best) [8,23]. The questionnaire was available in both Arabic and English versions and took approximately 10 min to complete (see Supplementary Materials for additional details). As per institutional guidelines, all study data is securely stored within encrypted folders and will be kept for five years.

For collecting data through an online questionnaire, many possible biases may emerge, such as selection bias, response bias, recall bias, nonresponse bias, duplicate responses, and measurement bias. Specific precautions were taken to avoid possible biases in data collection, such as using a validated tool, clear instructions, pilot testing, and assurances to the participants about anonymity and confidentiality. The Google Form option was used to avoid duplicate responses, allowing a respondent to fill out the questionnaire only once. Several biases that have been mentioned in the limitations cannot be controlled.

### 2.3. Statistical Analysis

A Microsoft Excel application was used to store the survey data, which was then processed using IBM SPSS Statistics 28 (Chicago, IL, USA) software. Descriptive statistics were presented for continuous variables (mean, standard deviation, median, and interquartile range, as applicable to the data distribution). In contrast, categorical variables were reported in terms of frequencies and percentages. The Shapiro–Wilk test was conducted to check normality for all continuous variables. ISQs, PMIF, WC:Ht and WC were normally distributed while height, weight, MBI, C index and WWI showed deviation from normal distribution. One-way ANOVA, followed by a post hoc test using Tukey’s HSD, or a chi-square test, followed by Bonferroni correction, was employed to evaluate the statistical differences in immune fitness variables between BMI groups using normal weight group as control, comparing it separately to the underweight, overweight and obese groups. Based on the distribution of the data, either the Mann–Whitney U-test (nonparametric distribution) or the independent *t*-test (normal distribution) was utilized to explore the statistical differences between the immune fitness groups. Correlations between anthropometric indices and ISQs and PMIF were assessed using Spearman’s or Pearson’s correlation coefficients, depending on the data distribution. *p*-values were adjusted using Bonferroni correction. Age and sex were included as covariates, and partial correlations were calculated to adjust for their effects. A multiple linear regression analysis was conducted to examine the association between anthropometric indices and IF, as measured by the ISQ score. Statistics were performed using two-tailed tests, with the statistical significance determined by a *p*-value of less than 0.05.

## 3. Results

In total, 1203 responses were collected. A total of 45 responses from those with a history of medical issues and/or those on immunosuppressive medication were excluded. Another 23 responses were excluded due to participants being under 18 years of age, leading to a final analysis of 1135 responses, of which 530 (46.7%) were males and 605 (53.3%) were females, with a mean age of 28.37 (±11.98). Among these, 478 (42.1%) had a normal BMI, and 343 (30.2%) were classified as overweight, 184 (16.2%) as obese, and 130 (11.5%) as underweight. Half of the individuals (51%) in the underweight group had an ISQ score <6 compared to 33.7% of individuals in the normal BMI group (*p* < 0.001). Similarly, 29.2% of individuals in the underweight group had a PMIF score of less than 6, compared to 11.9% of individuals in the normal BMI group (*p* < 0.001) (Table 1).

Allocating subjects into two cohorts based on their ISQ score revealed statistically significant differences in anthropometric parameters (weight, height, C index, and WWI) across ISQ groups (*p* < 0.05). Additionally, among individuals in the underweight group, a significantly higher proportion had an ISQ score of less than 6 (*p* < 0.001). More individuals in the high-WWI (*p* = 0.035) and high-C-index (*p* = 0.037) categories had reduced immune functioning (ISQ score < 6) compared to individuals with ISQ score ≥ 6 (Table 2).

A significant proportion of underweight and obese individuals scored fewer than six on past-year IF compared to those who scored more than six (*p* < 0.001). Furthermore, a notable proportion of individuals with a normal BMI scored more than six on the past-year IF score compared to those with a score of fewer than six (*p* < 0.001). Moreover, shorter individuals had scores of fewer than six on the past-year IF compared to their taller counterparts (*p* < 0.001) (Table 3).

A significant proportion of the underweight and obese individuals had momentary perceived immune fitness scores of less than 6 compared to those who had momentary perceived immune fitness scores of more than 6 (*p* < 0.001). Additionally, a significant proportion of the normal-BMI individuals had momentary perceived immune fitness scores of more than 6 compared to those who had momentary perceived immune fitness scores of less than 6 (*p* < 0.001).

**Figure 1:**
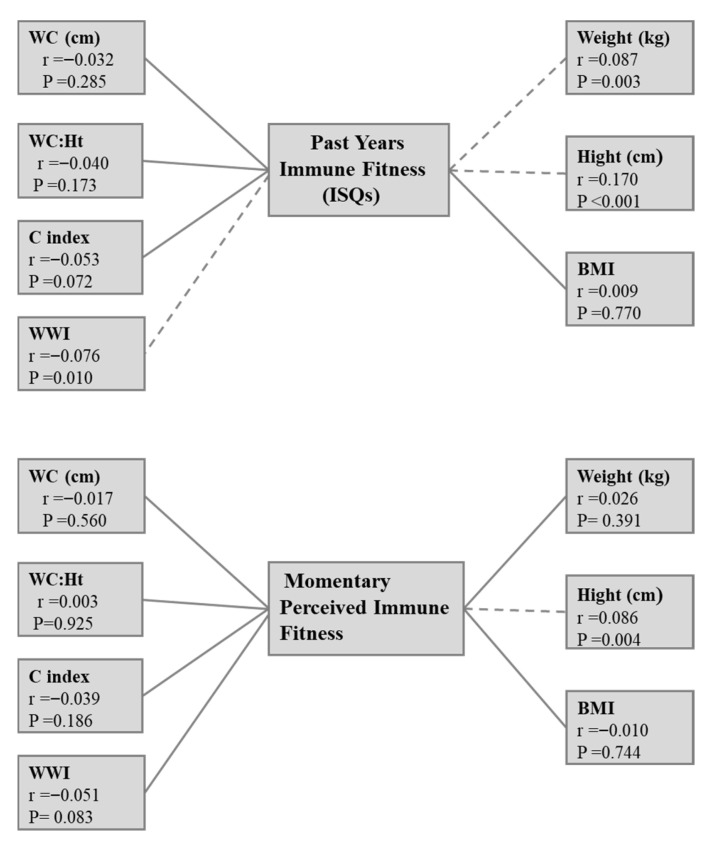
Associations between immune functioning and simple anthropometric indicators of obesity controlled for age and sex. Significant associations (*p* < 0.05) are depicted as interrupted lines.

After partial correlation controlling for age and sex, weight (*p* = 0.003), height (*p* < 0.001), and WWI (*p* = 0.01) were found to have significant correlations with past-year IF. In contrast, only height (*p* = 0.004) showed a significant correlation with PMIF (Figure 1).

A multiple linear regression analysis was performed to examine the association between various anthropometric indicators and IF, measured by the ISQ score (Table 4). The overall model was statistically significant. F (14,1118) = 4.86 and *p* < 0.001, explaining approximately 5.7% of the variance in immune fitness (R^2^ = 0.057, Adjusted R^2^ = 0.046). The predictors included waist-to-height ratio, age categories, weight (in kilograms), sex, waist circumference category, height (in centimeters), BMI category, conicity index, and weight-to-waist index categories. Significant predictors of immune fitness included sex (β = 0.067, 95% CI [0.05–0.714], *p* = 0.024), height (β = 0.177, 95% CI [0.019, 0.10], *p* < 0.001), and high-risk WC categories (β = −0.142, 95% CI [0.101, 1.554], *p* = 0.026). Specifically, males had higher IF scores compared to females. Higher scores are typically reported by taller individuals. However, those in the high-risk WC category had significantly lower IF scores than those in the low-risk group. In particular, men’s IF scores were, on average, 0.067 units higher than women’s. The IF score increased by 0.177 units for every 1 cm increase in height. On the other hand, the IF score decreased by 0.142 units for those in the high-risk WC category. Other predictors, such as BMI category, conicity index, WWI categories, waist-to-height ratio, weight, and age categories, were not statistically significant

## 4. Discussion

Our findings indicate that individuals classified as overweight or obese based on BMI do not significantly differ from those with a healthy body weight in terms of perceived IF, as assessed by ISQ score or the PMIF scale. The only notable difference is that a significantly higher percentage of obese participants scored below six on the PMIF scale compared to those with normal weight. However, the relatively small number of participants with a PMIF score below 6 in the normal-weight group limits the reliability of comparisons with other BMI subgroups. This contrasts with the findings for underweight individuals, who showed significant differences in perceived immune fitness. This aligns with recent findings showing that undernutrition impairs both innate and adaptive immunity due to deficiencies in key micronutrients such as vitamins A, D, and zinc [45,46]. It also compromises mucosal barriers and immune cell function, thereby increasing the risk of infection [47]. These effects may contribute to the subjective sense of reduced IF seen in underweight groups [8]. Nevertheless, given the cross-sectional design, these results are observational and do not establish causality. It is still possible that lower immune fitness may also contribute to underweight status.

Interestingly, the results of the current study align with the only other study comparing perceived IF across BMI categories, which found no significant differences in perceived IF between individuals with a normal weight and those in the overweight and obesity class I and II categories. However, similar to our study, that research also reported significantly lower PMIF in those who are overweight or obese compared to the normal-weight group [8].

The absence of significant variations in BMI > 25 across IF subgroups in the current study, coupled with the lack of a significant correlation with perceived IF, suggests that alternative anthropometric measures of obesity may be more sensitive indicators of perceived immune fitness. Consistent with this, a previous study conducted on Saudi females demonstrated that WC is the best measure of adiposity, showing more significant differences in immune system markers and higher inflammation, evidenced by elevated total and specific leukocyte counts and CRP concentrations, compared to BMI [48]. Similarly, another recent study found that, among the various tested anthropometric measures, WC showed the strongest association with white blood cell count and high-sensitivity C-reactive protein [49].

Identifying anthropometric measures that effectively capture obesity-related reduced IF is essential for targeting high-risk groups for intervention. In this context, the current analysis of anthropometric indicators across ISQ groups revealed significant differences between individuals with poor immune status and those with good immune status. Specifically, those with poor immune status exhibited significant variations in weight, height, C-index, WWI, and waist-to-height ratio, also showing a trend toward significance, which highlights the potential of these measures in identifying individuals at greater risk of poor immune function (IF). Moreover, regression analysis further identified sex, height, and waist circumference categories as significant predictors of IF. It is essential to note that the location of body fat is closely linked to immune dysfunction, which is more prevalent in visceral obesity than in subcutaneous obesity [50,51]. This may explain why indicators of central obesity, such as WC-to-height ratio, C-index, and WWI, were associated with poor IF. Similar results were reported by few other studies [49,50,51]. Similar to the present study, the weight-adjusted waist index (WWI) was found to be superior to both BMI and waist circumference [52]. The clinical relevance of this finding lies in the direct link between fat distribution patterns and morbidity and mortality in adults, with particular emphasis on the strong correlation to visceral fat mass [53,54]. Some lean individuals can experience immune-metabolic disturbances similar to those seen in obese individuals, primarily due to a similar fat distribution pattern [53]. Additionally, obese individuals without visceral obesity are often referred to as ‘metabolically healthy obese,’ as they do not exhibit immune-metabolic disturbances at a specific point in time. However, metabolically healthy obesity should not be considered a benign condition that does not require treatment; instead, it can guide decision-making for a more personalized and risk-based approach to obesity management [55]. Obesity leads to a shift in the immune profile toward a pro-inflammatory state characterized by the recruitment of macrophages, neutrophils, and cytotoxic CD8+ T cells. This shift is driven by pro-inflammatory factors released by adipocytes, along with conditions like hypoxia and adipocyte cell death, which promote further low-grade inflammation [50,56]. Recent research has shown that persistent insulin resistance linked to obesity in our current lifestyle promotes low-grade inflammation, as well as metabolic, immune, and allergic disorders [57]. Observations from diet-induced obesity mouse models demonstrate that obesity impairs the function of tumor-infiltrating CD8+ T cells, increasing cancer incidence. Food restriction, on the other hand, restores immune function and improves the incidence of cancer [58]. Chronic inflammation caused by obesity alters the composition and function of immune cells, increasing pro-inflammatory neutrophils, macrophages, and T cells, and decreasing anti-inflammatory macrophages and mesenchymal stem cells. A dysregulated immune response impairs local tissue repair, as demonstrated by a decrease in bone formation around titanium implants in obese mice, and is induced by systemic changes in circulating adipokines and cytokines [59]. In light of these findings, it is highly recommended to move beyond BMI when assessing obesity risk.

In terms of PMIF, the current study showed no correlation between anthropometric parameters, except for height and the scores, even after controlling for age and sex. These results may be attributed to the nature of the question on PMIF being ratable, subjective, and dependent on the participants’ self-belief and awareness, unlike the questions in the ISQ, which were more objective and based on specific, measurable symptoms.

The modest explanatory power of the model (4.6%) reflects the multifactorial nature of immune fitness, influenced by genetic, environmental, and lifestyle factors beyond anthropometry. This is not unexpected, as these factors were not captured in the present model. However, this study highlights the necessity of including multiple anthropometric measures rather than relying solely on BMI when investigating obesity and immune function.

The present study’s findings indicate the clinical significance of the waist circumference, height, and sex in assessing IF. It seems that taking into consideration alternative anthropometric indices, such as the weight-adjusted waist index (WWI) and C index, may contribute to the early identification of individuals with reduced IF. The association of IF with these parameters will help clinicians to plan personalized interventions targeting body composition and lifestyle factors that can alleviate IF, disease prevention, and overall health outcomes among different population groups.

Several limitations are present in this study. Its cross-sectional observational approach precluded the assessment of causality and temporal effects, and we were unable to exclude residual confounding effects. Second, although the questionnaire clearly explained how to take the measurements, the self-reported anthropometrics may still be underestimated or overstated. The study’s conclusions may only be applicable to the Makkah region due to the selection bias created by the convenience sample and the online self-administered questionnaire. Study participants were subject to recall bias and social desirability effects in the survey. Additionally, many confounders that can influence the results, such as physical activity, dietary habits, chronic diseases, smoking and alcohol consumption, sleep quality and stress, and socioeconomic status, could not be assessed. Few correlations were marginally significant, so conclusion should be drawn cautiously.

Despite these drawbacks, this study is the first to investigate the association between anthropometric measures and immune fitness, as evaluated by the ISQ, to help facilitate the development of more effective interventions aimed at optimizing immune health across diverse populations and promoting the immune resilience of society in preparation for any potential health crisis. An evaluation of the association between clinical measures of immune competence (objective), ISQ scores (subjective), and anthropometric measurements should be conducted in large-scale populations to verify the correlation observed in the present study.

Clinically, these results underscore the necessity of not relying exclusively on BMI since it might not accurately reflect body fat distribution, particularly central obesity, which is more strongly associated with inflammatory [60] and metabolic risks [61]. By integrating multiple anthropometric indices, body fat and its distribution may be more accurately assessed, making it easier to identify and stratify individuals at a higher risk of obesity-related complications. Nevertheless, these results should be interpreted cautiously given the study limitation.

## 5. Conclusions

These results give the idea that additional anthropometric measures, particularly those reflecting fat distribution, may contribute to a more comprehensive understanding of how body composition relates to IF. This highlights the need to move beyond traditional measures of obesity and consider alternative indicators, such as those reflecting central adiposity, when evaluating potential associations with immune fitness. It is suggested that moving beyond simple weight-based classifications to include diverse anthropometric indicators is important for public health and clinical practice. This approach can support the development of more effective interventions aimed at optimizing immune health across populations and enhancing societal resilience against potential health crises. Future research integrating immune biomarkers and ISQ assessments with anthropometric data would provide valuable insights. Additionally, given the relatively young mean age of the study cohort and the recruitment from a single region, the findings of this study may not be generalizable to other age groups or to populations from other regions of Saudi Arabia. Including more heterogeneous age groups and broader geographic representation in future studies are warranted to validate and extend these observations.

## Figures and Tables

**Table 1:** Immune status score and momentary perceived immune fitness scores across different BMI groups.

BMI	Underweight	Normal Weight	Overweight	Obese	Total	*p*-Value
**N (%)**	130 (11.5)	478 (42.1)	343 (30.2)	184 (16.2)	1135 (100)	
**Gender**						
Male	68 (12.8)	235 (44.3)	151 (28.5)	76 (14.3)	530 (100)	
Female	62 (10.2)	243 (40.2)	192 (31.7)	108 (17.9)	605 (100)	
**Past year immune fitness (ISQ) score**						
Score	5.68 (±3.03) *	6.78 (±2.83)	6.83 (±2.77)	6.23 (±2.75)	6.59 (±2.84)	<0.001
% <6	67 (51.5) *	161 (33.7)	115 (33.5)	74 (40.2)	417 (36.7)	<0.001
**Momentary perceived immune fitness**						
Score	6.98 * (±2.57)	7.83 (±2.1)	7.77 (±2.1)	7.48 (±2.2)	7.66 (±2.17)	<0.001
% <6	38 (29.2) * ^a^	57 (11.9)	53 (15.5)	40 (21.7) *^b^	188 (16.6)	<0.001 ^a^ 0.008 ^b^

**Note**: Data reported as median (IQR), mean (±SD) or number (%). Test used = one way ANOVA followed by post hoc test Tuky HSD or chi square test followed by Bonferroni correction. Differences from normal weight group are statistically significant if *p* < 0.05 (indicated by *). Specifically, ‘^a^’ indicates the underweight group differs from the normal-weight group, and ‘^b^’ indicates the obese group differs from the normal-weight group.

**Table 2:** Distribution of anthropometric indicators according to past-year immune fitness scores.

Variables	Total N = 1135	ISQ Score Below Cut-Off Value (ISQ < 6) N = 417 (36.75)	ISQ Score Above Cut-Off Value (ISQ ≥ 6) N = 718 (63.25)	*p*-Value
**Age**				
18–29	762 (67.2%)	289 (69.6%)	473 (65.8%)	0.39
30–44	214 (18.9%)	73 (17.1%)	143 (19.9%)	
>45	158 (13.9%)	55 (13.3%)	103 (14.3%)	
**Weight (kg)**	66 (25)	64 (30)	68 (24)	0.001
**Height (cm)**	164 (15)	161 (14)	166 (15)	<0.001
**BMI categories**				
Underweight	130 (11.5%)	67 (16.1%) ^a^	63 (8.8%) ^b^	<0.001
Normal	478 (42.1%)	161 (36.8%) ^a^	317 (44.2%) ^a^	
Overweight	343 (30.2%)	115 (27.6) ^a^	228 (31.8%) ^a^	
Obese	184 (16.2%)	74 (17.7%) ^a^	110 (15.3%) ^a^	
**WC (cm)**				
Low risk	371 (32.7%)	130 (31.2%)	241(33.5%)	0.20
Moderate risk	316 (27.8%)	129 (30.9%)	187 (26%)	
High risk	449 (39.5%)	158 (37.9%)	291 (40.5%)	
**WC:Ht**	0.58 (±1.5)	0.67 (±2.5)	0.53 (±0.14)	0.068
**C index**	1.24 (0.42)	1.28 (0.45)	1.23 (0.40)	0.037
**WWI**				
Low	465 (40.9%)	156 (37.4%) ^a^	309 (43%) ^a^	0.035
Medium	172 (15.1%)	57 (13.7%) ^a^	115 (16%) ^a^	
High	499 (43.9%)	204 (48.9%) ^a^	295 (41%) ^b^	

**Note**: Data are reported as median (IQR), mean (± SD), or number (%). Test used: Mann–Whitney U-test, student *t* test, Chi-square test with Bonferroni correction. Differences are significant if *p* < 0.05. **Abbreviations:** ISQ—Immune Status Questionnaire; BMI, body mass index; WC, waist circumference; Ht, height; C index, conicity index; WWI, weight-adjusted waist index. **Used cut-offs:** WC: men—low risk <94 cm, moderate risk 94–101.9 cm, high risk ≥102 cm; women—low risk <80 cm, moderate risk 80–87.9 cm, high risk ≥88 cm. WWI: low risk <10, moderate risk 10–11, high risk >11. Superscript letters indicate the pairwise differences between groups; only those with different superscript letter are statistically significant.

**Table 3:** Distribution of anthropometric indicators according to momentary perceived immune fitness scores.

Variables	Total N = 1135	Below Cut-Off Value (ISQ < 6) N = 187 (16.6%)	Above Cut-Off Value (ISQ ≥ 6) N = 949 (83.4%)	*p*-Value
**Age**				0.437
18–29	762 (67.2%)	131 (70.1%)	633 (66.6%)	
30–44	214 (18.9%)	29 (15.5%)	185 (19.5%)	
>45	158 (13.9%)	27 (14.4%)	131 (13.8%)	
**Weight (kg)**	66 (25)	64.5 (30)	66 (24)	0.423
**Height (cm)**	164 (15)	160 (15)	165 (14)	<0.001
**BMI (kg/m^2^)**				
Underweight	130 (11.5%)	38 (20.2%)	92 (9.7%) *	<0.001
Normal	478 (42.1%)	57 (30.3%)	421 (44.5%) *	
Overweight	343 (30.2%)	53 (27.2%)	290 (30.6%)	
Obese	184 (16.2%)	40 (21.3%)	144 (15.2%) *	
**WC categories**				0.220
Low risk	371 (32.7%)	58 (30.9%)	313 (33%)	
Moderate risk	316 (27.8%)	62 (33%)	254 (26.8%)	
High risk	449 (39.5%)	68 (36.2%)	381 (40.2%)	
**WC:Ht**	0.58 (±1.5)	0.54 (±0.137)	0.59 (±1.65)	0.689
**C index**	1.24 (0.42)	1.27 (0.41)	1.24 (0.42)	0.950
**WWI categories**				
Low	465 (40.9%)	79 (42%)	386 (40.7%)	0.159
Medium	172 (15.1%)	20 (10.6%)	152 (16%)	
High	499 (43.9%)	89 (47.3.8%)	419 (43.2%)	

**Note**: Data are reported as median (IQR), mean (± SD), or number (%). Test used: Mann–Whitney U-test, student *t* test, Chi-square test with Bonferroni correction. Differences are significant if *p* < 0.05. Asterisks denote significant differences in the proportion of individuals with ISQ scores <6 versus ≥6 among the underweight, normal, and obese groups. **Abbreviations:** ISQ—Immune Status Questionnaire; BMI, body mass index; WC, waist circumference; Ht, height; C index, conicity index; WWI, weight-adjusted waist index. **Used cut-offs:** WC: men—low risk <94 cm, moderate risk 94–101.9 cm, high risk ≥102 cm; women—low risk <80 cm, moderate risk 80–87.9 cm, high risk ≥88 cm. WWI: low risk <10, moderate risk 10–11, high risk >11.

**Table 4:** Multiple linear regression analysis of the association between anthropometric indicators and ISQ score.

Variable	B	SE B	β	95% CI		*p*-Value
				**LL**	**UL**	
Constant	1.06	1.535	—	−1.953	4.072	0.49
**Sex**						
Female (reference)						
Male	0.41	0.169	0.067	0.05	0.714	0.024
Weight (kg)	−0.006	0.007	−0.05	−0.021	0.008	0.39
Height (cm)	0.04	0.01	0.177	0.019	0.1	<0.001
WC:Ht	0.063	0.063	0.033	−0.06	0.185	0.317
C Index	−0.81	0.47	−0.102	−1.502	0.72	0.059
**BMI category**						
Normal (reference)						
Underweight	−0.827	0.3	−0.93	−1.414	−0.239	0.006
Overweight	−0.099	0.235	−0.16	−0.56	0.361	0.672
Obese	−0.627	0.371	−0.81	−1.354	0.101	0.091
**WC category**						
Low risk (reference)						
Moderate risk	−0.19	0.257	−0.03	−0.314	0.694	0.46
High risk	−0.827	0.37	−0.142	0.101	1.554	0.026
**WWI Category**						
Low risk (reference)						
Moderate risk	−0.163	0.291	−0.021	−0.733	0.408	0.576
High risk	−0.433	0.355	−0.076	−1.131	0.264	0.223
**Age Categories**						
18–29 (reference)						
30–44	0.209	0.218	0.029	−0.218	0.636	0.338
>45	0.394	0.247	0.048	−0.091	0.88	0.111

**Model statistics:** R = 0.24, R^2^ = 0.057, Adjusted R^2^ = 0.046, F (14,1118) = 4.86, *p* < 0.001. **Abbreviations:** BMI, body mass index; WC, waist circumference; Ht, height; C index, conicity index; WWI, weight-adjusted waist index, CI, confidence interval; LL, lower limit of CI, UL, upper limit of CI. **Used cut-offs:** WC: men—low risk <94 cm, moderate risk 94–101.9 cm, high risk ≥102 cm; women—low risk <80 cm, moderate risk 80–87.9 cm, high risk ≥88 cm. WWI: low risk <10, moderate risk 10–11, high risk >11.

## Data Availability

The original contributions presented in this study are included in the article/Supplementary Material. Further inquiries can be directed to the corresponding author.

## Supplementary Materials

The following supporting information can be downloaded at https://www.mdpi.com/article/10.3390/healthcare14081046/s1. File S1: Integrated Consent Form and Questionnaire (Arabic–English).
