# Supplementary material for: Anthropometric Indicators and Immune Fitness: An Exploratory Online Survey Among Adults from Saudi Arabia
Source: Healthcare (Basel). 2026 Apr 15;14(8):1046. doi: 10.3390/healthcare14081046 (PMC13115780; doi:10.3390/healthcare14081046)
Supplement: Supplementary file 1 [file healthcare-14-01046-s001.zip › healthcare-4171136-supplementary.pdf]

You are invited to participate in a study titled:

“Anthropometric indicators and immune fitness: an exploratory online survey among Adults from Saudi Arabia”

This study is being conducted by **Dr. Azzah S. Alharbi**, Faculty of Medicine, Department of clinical Microbiology and immunology, King Abdulaziz University.

Purpose of the Study:
To assess the status of adults' immune fitness and explore the association between immune fitness and anthropometric indicators of obesity that may affect immune function.

Participation:
Your participation is voluntary. You may refuse to take part or exit the survey at any time without penalty. You may skip any question you do not wish to answer. Participation should take approximately **10 minutes**.

Benefits & Risks:
You will receive no direct benefits. Your responses may help us understand medical students’ immune fitness. There are no foreseeable risks beyond those encountered in daily life.

Confidentiality:
Your participation is completely anonymous. No information you share can be traced electronically to you or your device. Responses will be stored in a **password-protected electronic format** accessible only by Dr. Azzah S. Alharbi.

Contact Information:

- **Dr. Azzah S. Alharbi** – asalharbi3@kau.edu.sa
- For rights as a participant, contact the Institutional Review Board: med.rcommittee@kau.edu.sa

Electronic Consent:
By clicking **“Agree”**, you confirm that you have read the above information, voluntarily agree to participate, are 18 years of age or older, and are eligible to proceed to the questionnaire.

- ☐ Agree
- ☐ Disagree

أنت مدعو للمشاركة في دراسة بعنوان

المؤشرات الجسمية المتعلقة بالسمنة واللياقة المناعية: مسح استكشافي عبر الإنترنت بين البالغين
"السعوديين **والمقيمين من منطقه مكة المكرمة**

تُجرى هذه الدراسة بواسطة **الدكتورة عزة الحربي**، كلية الطب، قسم علم الأحياء الدقيقة والمناعة السريرية، جامعة الملك عبد العزيز.

هدف الدراسة
تقييم حالة اللياقة المناعية للبالغين السعوديين والمقيمين في منطقه مكة المكرمة واستكشاف العلاقة بين اللياقة المناعية و مختلف المؤشرات الجسمية للسمنة والتي قد تؤثر على وظيفة الجهاز المناعي.

المشاركة
مشاركتك طوعية. يمكنك رفض المشاركة أو الخروج من الاستبيان في أي وقت دون أي عقوبة. يمكنك تخطي أي سؤال لا ترغب بالإجابة عليه. من المتوقع أن تستغرق المشاركة اقل من ١٠ دقائق

الفوائد والمخاطر
لن تحصل على أي فوائد مباشرة، ولكن قد تساعد إجاباتك في فهم حالة اللياقة المناعية لدى طلاب الطب. لا توجد مخاطر متوقعة بخلاف تلك التي تواجهها في حياتك اليومية.

السرية
مشاركتك مجهولة تمامًا. لا يمكن تتبع أي معلومات تقدمها إليك أو إلى جهازك إلكترونيًا. سيتم حفظ الإجابات في **ملف إلكتروني محمي بكلمة مرور**، ولن يكون الوصول إليه إلا للدكتورة عزة س. الحربي.

معلومات التواصل

- **الدكتورة عزة س. الحربي**– asalharbi3@kau.edu.sa
- لحقوقك كمشارك، تواصل مع لجنة الأخلاقيات المؤسسية: med.rcommittee@kau.edu.sa

:الموافقة الإلكترونية
اذا كنت تبلغ من العمر ١٨ عاما فاكثر ، فإنك تؤكد أنك قرأت المعلومات أعلاه، وتوافق طواعية على المشاركة، وأنك مؤهل للانتقال إلى الاستبيان

- ☐ موافق
- ☐ غير موافق

Questionnaire / الاستبيان

1. **Age:------------**
2. **Nationality**
   - Saudi / سعودي
   - Non saudi / غير سعودي
3. **Gender / الجنس**
   - Male/ ذكر
   - Female/انثى
4. **Are you a resident of the Makkah Region (Western Region)?/ هل انت مقيم في منطقه مكة المكرمة (المنطقة الغربية)**
   - Yes/ نعم
   - No/ لا

5. Body Measurements / القياسات الجسدية

Height / الطول بالمتر (m): ________

Weight / الوزن بالكيلوغرام (kg): ________

Waist circumference / محيط الخصر بالسم: ________

To measure your waist circumference accurately as shown in figure :

1. Find the top edge of your hip bone (iliac crest).
2. Find the bottom edge of your ribs (ribcage).
3. Measure the distance midway between these two points. (This is where your waist circumference should be measured- red line in figure)
4. Keep the tape measure but not tight, and ensure it is horizontal all around your waist.
5. Record the measurement in centimeters.

لقياس محيط الخصر بدقة كما هو موضح بالصورة :

أولا حدد أعلى حافة عظم الورك (الحافة الحرقفية) ثم حدد أسفل حافة القفص الصدري وانت واقفا

ثانيا قس المحيط **في منتصف المسافة بين هاتين النقطتين** (هذا هو المكان الصحيح لقياس محيط الخصر المحدد بالخط الأحمر في الصورة)

ثالثا ضع شريط القياس بشكل مريح دون شد مفرط، وتأكد أن يكون أفقيًا حول الخصر و سجل القياس بالسنتيمتر.

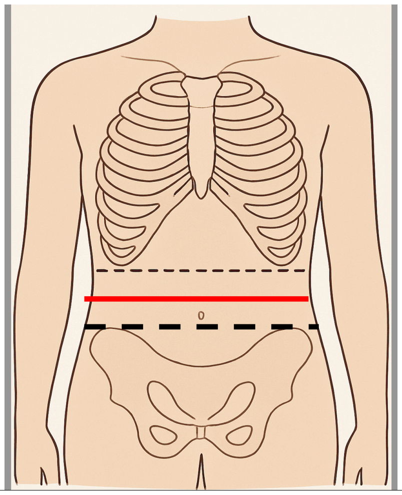

---

6. Have you ever been diagnosed with any of the following conditions? / هل تم تشخيصك باي من الحالات التالية؟

- ☐ **Immune disease / أمراض مناعية**
  (e.g., lupus, rheumatoid arthritis, multiple sclerosis, immundefeciency / مثل: الذئبة،مرض نقص المناعه، التهاب المفاصل الروماتويدي، التصلب المتعدد)
- ☐ **Chronic disease / أمراض مزمنة**
  (e.g., , heart disease, liver disease chronic kidney disease, cancer / مثل: ، ، امراض الكبد الأورام ، الفشل الكلوي المزمن أمراض القلب)

- ☐ **Taking immunosuppressant drugs / أتناول أدوية مثبطة للمناعة**
  (e.g., corticosteroids, biologics, chemotherapy، / مثل: كورتيزون طويل الأمد، أدوية بيولوجية، العلاج الكيميائي)
- ☐ *Pregnancy (حمل)*
- ☐ **None of the above / لا شيء مما سبق**
- **Other, pleses spesify/ أخرى يرجى ذكرها**

---

7. How often you have had any of the following sysmpotms in the past 12 months

هل اصبت باي من الاعراض التالية خلال السنة الماضية (١٢ شهرا) /

1. **Common cold / نزلات البرد**

- ☐ Never / أبداً
- ☐ Sometimes / أحياناً
- ☐ Regularly / بشكل منتظم
- ☐ Often / غالباً
- ☐ Always / دائماً

2. **Headache / صداع**

- ☐ Never / أبداً
- ☐ Sometimes / أحياناً
- ☐ Regularly / بشكل منتظم
- ☐ Often / غالباً
- ☐ Always / دائماً

3. **Muscle and joint pain / آلام العضلات والمفاصل**

- ☐ Never / أبداً
- ☐ Sometimes / أحياناً
- ☐ Regularly / بشكل منتظم
- ☐ Often / غالباً
- ☐ Always / دائماً

4. **Diarrhea / إسهال**

- ☐ Never / أبداً
- ☐ Sometimes / أحياناً
- ☐ Regularly / بشكل منتظم

- ☐ Often / غالباً
- ☐ Always / دائماً

5. **Skin problems (acne, eczema) / مشاكل جلدية (حبوب الشباب – أكزيما)**

- ☐ Never / أبداً
- ☐ Sometimes / أحياناً
- ☐ Regularly / بشكل منتظم
- ☐ Often / غالباً
- ☐ Always / دائماً

I score my general health from 0 to 10 / كيف تقيم صحتك العامة من ٠ الي ١٠
0 = very bad / سيء جداً

10 = very good / ممتاز

I score my immune functioning from 0 to 10 / كيف تقيم كفاءة جهازك المناعي من ٠ الي ١٠
0 = very bad / سيء جداً

10 = very good / ممتاز

Thank you for your participation! / شكرًا لمشاركتكم!
